# Hexons from adenovirus serotypes 5 and 48 differentially protect adenovirus vectors from neutralization by mouse and human serum

**DOI:** 10.1371/journal.pone.0192353

**Published:** 2018-02-05

**Authors:** Andrew W. Harmon, Rituparna Moitra, Zhili Xu, Andrew P. Byrnes

**Affiliations:** Division of Cellular and Gene Therapies, Center for Biologics Evaluation and Research, Food and Drug Administration, Silver Spring, Maryland, United States of America; Northwestren University, UNITED STATES

## Abstract

Adenovirus vectors are widely used in gene therapy clinical trials, and preclinical studies with these vectors are often conducted in mice. It is therefore critical to understand whether mouse studies adequately predict the behavior of adenovirus vectors in humans. The most commonly-used adenovirus vectors are derived from adenovirus serotype 5 (Ad5). The Ad5 hexon protein can bind coagulation factor X (FX), and binding of FX has a major impact on vector interactions with other blood proteins. In mouse serum, FX protects Ad5 vectors from neutralization by natural antibodies and complement. In the current study, we similarly find that human FX inhibits neutralization of Ad5 vectors by human serum, and this finding is consistent among individual human sera. We show that human IgM and human IgG can each induce complement-mediated neutralization when Ad5 vectors are not protected by FX. Although mouse and human serum had similar effects on Ad5 vectors, we found that this was not true for a chimeric Ad5 vector that incorporated hexon regions from adenovirus serotype 48. Interestingly, this hexon-chimeric vector was neutralized by human serum, but not by mouse serum. These findings indicate that studies in mouse serum accurately predict the behavior of Ad5 vectors in human serum, but mouse serum is not an accurate model system for all adenovirus vectors.

## Introduction

Adenovirus (Ad) vectors are one of the most popular vectors for gene therapy and have been used in roughly 20% of gene therapy clinical trials to date [1]. When administered intravenously (i.v.), Ad vectors have the potential to reach any vascularized organ or tumor, but their biodistribution has proven difficult to control. Poor targeting of vectors not only reduces the efficiency of gene therapy but can also increase risks, for example when vectors cause liver damage because of undesired targeting to hepatocytes [2]. Although vector biodistribution is influenced by the expression pattern of viral receptors, it has become increasingly clear that host blood proteins have an equally important influence on vector biodistribution [3, 4].

When Ad serotype 5 (Ad5) vectors are administered i.v., interaction of vector with coagulation factor X (FX) is a key determinant of liver biodistribution. The Ad5 hexon trimer has a high affinity binding site for FX, and Ad5 vectors only transduce hepatocytes efficiently *in vivo* when the hexon protein can bind FX [5–7]. This critical role for FX in liver transduction by Ad5 vectors has been demonstrated in mice, rats and non-human primates [5, 8]. Although it was initially thought that promotion of liver transduction by FX was due to the ability of FX to enhance vector binding to heparan sulfate, recent work has shown that liver heparan sulfate is not essential for liver transduction by Ad5 vectors [9]. Instead, it is now apparent that the major impact of FX on Ad5 vectors *in vivo* is due to the ability of FX to protect Ad5 from inactivation by other proteins in the blood, specifically natural antibodies and complement [10].

Natural antibodies are germline-encoded antibodies that possess unusually long and flexible variable regions. These unique variable regions allow natural IgM antibodies to be polyreactive: each antibody can bind with low affinity to multiple different antigens [11, 12]. Although each individual Fab-antigen interaction of this type may be weak, IgM antibodies are pentameric or hexameric and thus have an increased ability to bind to repetitive structures such as viral capsids [13, 14]. Natural IgM can bind to Ad vectors, and one consequence is that IgM enhances Ad vector clearance from the circulation by Kupffer cells and thereby reduces liver transduction [15–18].

Multivalent binding of IgM to an antigen causes a conformational change in IgM that allows it to activate the classical complement pathway [19–21]. It is therefore intriguing that Ad5 vectors normally fail to activate complement in mouse serum, even though Ad5 vectors are able to bind IgM [10]. However, when FX is blocked or when hexon is mutated so that the vector is unable to bind FX, Ad5 vectors become strong activators of complement in mouse serum and become neutralized by mouse serum in a process that depends on both IgM and complement [10, 22]. FX also plays a protective role in other rodent sera, protecting Ad5 from neutralization by guinea pig and rat sera [10, 22]. When Ad5 vectors are administered i.v., FX is essential for liver transduction in wild-type mice, but FX is not needed for liver transduction in mouse strains that lack antibodies or complement [10, 22]. Thus, the major impact of FX on Ad5 vectors both *in vitro* and *in vivo* is through an ability of FX to protect Ad5 vectors from being attacked by complement.

The current study investigates whether Ad vectors behave similarly in mouse and human serum, with the goal of understanding the predictive value of Ad gene therapy studies in mice. We show here that FX protects Ad5 vectors from neutralization by complement in both mouse and human serum. Interestingly, however, we find that a chimeric Ad5 vector with a modified hexon based on Ad48 is neutralized by human serum, but not by mouse serum, which indicates that there are major species-dependent differences in neutralization for some Ad vectors.

## Results

### FX inhibits neutralization of Ad5 in human serum

In mouse serum, FX protects Ad5 vectors from neutralization by natural antibodies and complement [10]. A major goal of the current study was to determine whether human FX similarly protects Ad5 from neutralization in human serum. One complicating factor when using human sera is that many humans have neutralizing IgG antibodies against Ad due to childhood Ad infections [23]. Therefore, our experiments focused on human sera with low levels of anti-Ad5 neutralizing antibodies, to provide the most straightforward model system and to allow comparison with our previous studies, which used serum from mice that had no prior exposure to Ad5.

**Fig 1:**
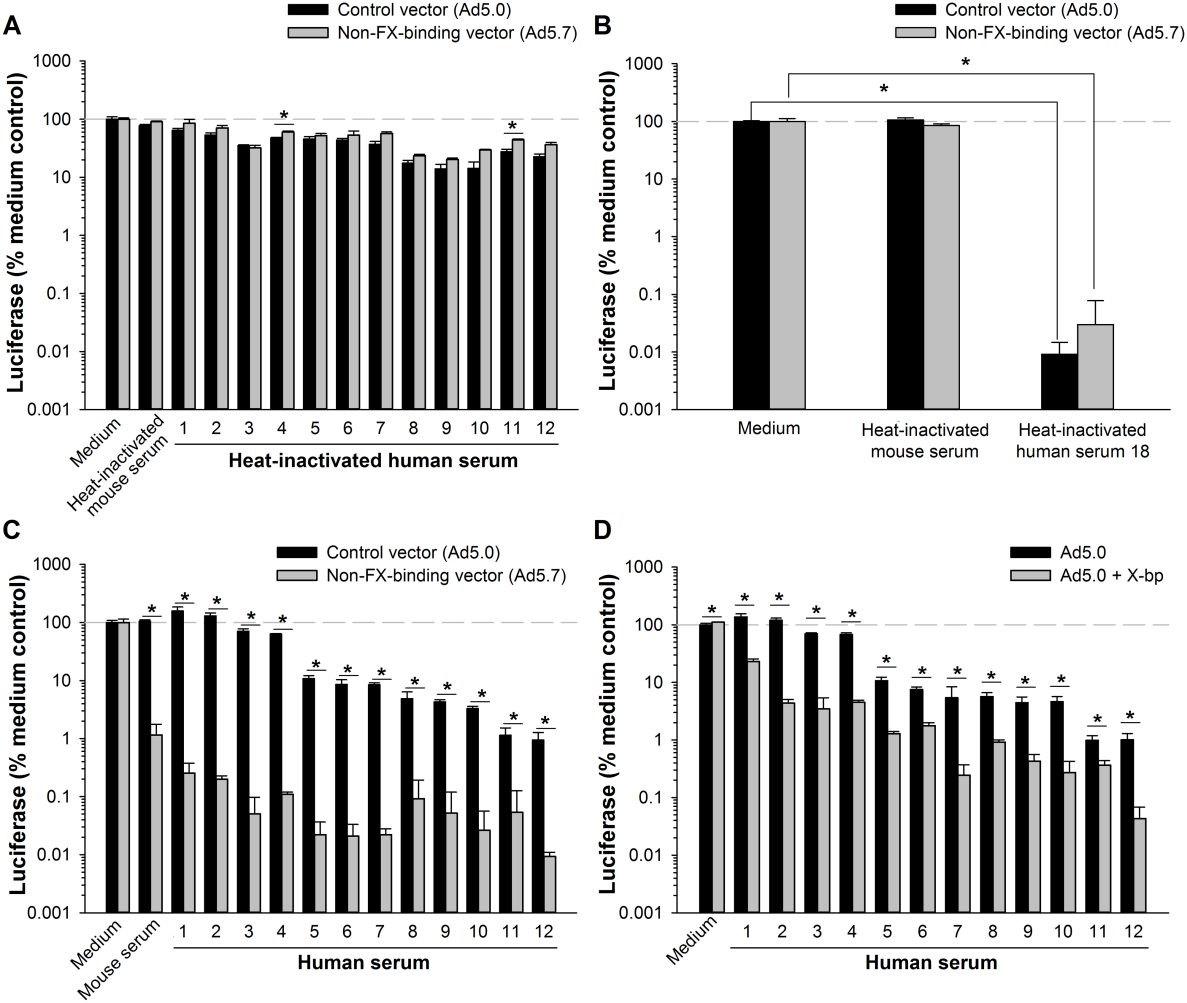
FX inhibits neutralization of Ad5 in human serum. Luciferase-expressing Ad5 vectors were incubated at 37°C with either C57BL/6 mouse serum or individual human sera at a serum concentration of 80%. Samples were then diluted and assayed for infectivity on 293 cells. (A) Infectivity of vectors after incubation with 12 human sera that have low antibody-mediated neutralizing activity. Sera were heat-inactivated at 56 °C to destroy complement. Ad5.0 is a control Ad5 vector with a wild-type capsid, and Ad5.7 is a modified vector that is unable to bind coagulation factors due to mutation of the FX binding site in hexon. (B) Neutralization of vectors by a human serum that has high antibody-mediated neutralizing activity (sample 18, heat-inactivated). (C) Effect of non-heat-inactivated serum on infectivity of Ad5.0 and Ad5.7 vectors. (D) Impact of blocking FX (with 80 μg/mL X-bp) on neutralization of Ad5.0 by non-heat-inactivated serum. T-tests were used to evaluate differences, followed by the sequential Holm-Sidak procedure for multiple comparisons to maintain the experimentwise type I error rate at ≤ 0.05. * Groups are significantly different (*p* ≤ 0.05). Each bar represents the mean of 3–4 replicates ± S.D.

Complement plays an essential role in neutralization of Ad5 vectors [10]; therefore, we obtained human sera from a commercial supplier that collects and stores sera in a manner that preserves complement activity. To screen for sera with low levels of anti-Ad5 neutralizing antibody, we heat-inactivated a sample of each serum (to destroy complement activity) and identified those that poorly neutralized Ad5 vectors. Samples were classified as having low antibody-mediated neutralizing activity if an 80% concentration of heat-inactivated serum did not inhibit vector infectivity by more than 90%. Of the 30 individual human sera that were screened, we classified 12 sera as having low antibody-mediated neutralizing activity under these conditions (Fig 1A). An example of a human serum sample with high antibody-mediated neutralizing activity (sample 18) is shown in Fig 1B.

To determine whether coagulation factors are able to protect Ad5 from neutralization by normal human serum (non-heat-inactivated serum having active complement), we compared the ability of sera to neutralize Ad5.0 (a normal Ad5 vector with a wild-type capsid) and Ad5.7 (a modified vector that cannot bind coagulation factors due to mutation of the FX binding site in hexon). All 12 of the human sera neutralized Ad5.7 to a significantly greater extent than they neutralized Ad5.0 (Fig 1C). When these same 12 sera were heat-inactivated, they failed to preferentially neutralize Ad5.7 (Fig 1A). These results indicate that coagulation factors protect Ad5 from neutralization by complement in human sera, just as coagulation factors protect Ad5 from neutralization in mouse sera.

Although we selected these 12 human sera because heat-inactivated sera had shown less than 90% neutralizing activity for Ad5.0 (Fig 1A), when these same sera had active complement (non-heat-inactivated) we found that many of these sera were able to neutralize Ad5.0 by more than 90% (Fig 1C). Thus, although coagulation factors provided a significant degree of protection from complement-mediated neutralization in all of these human sera (as evidenced by lower neutralization of Ad5.0 relative to Ad5.7), coagulation factors do not provide complete protection against neutralization by complement in all cases.

A snake venom protein, X-bp [24], binds and inhibits both mouse FX and human FX [5]. We used X-bp to confirm that human FX protects Ad5 from neutralization by human serum. Addition of X-bp led to significantly more neutralization of Ad5.0 by all 12 of the human sera (Fig 1D), indicating that human FX inhibits neutralization of Ad5 by human serum. We noted that neutralization of Ad5.0 in the presence of X-bp was not as extensive as neutralization of Ad5.7 (compare Fig 1C and 1D). We speculate that X-bp may not be able to block all of the FX in the serum, even at the maximally feasible concentration of X-bp that we used here.

Thus, we established a critical protective role for FX in human sera using two independent methods to prevent FX from interacting with Ad5: mutating the vector hexon protein and inhibiting FX directly with X-bp. Protection by FX in individual human sera was always observed; every one of the 12 tested human sera showed enhanced neutralizing activity when FX was prevented from interacting with the vector. Taken together, these results indicate that FX protects Ad5 from complement-mediated neutralization in both mouse and human serum.

### Human IgM binds to Ad5, but is insufficient for neutralization

**Fig 2:**
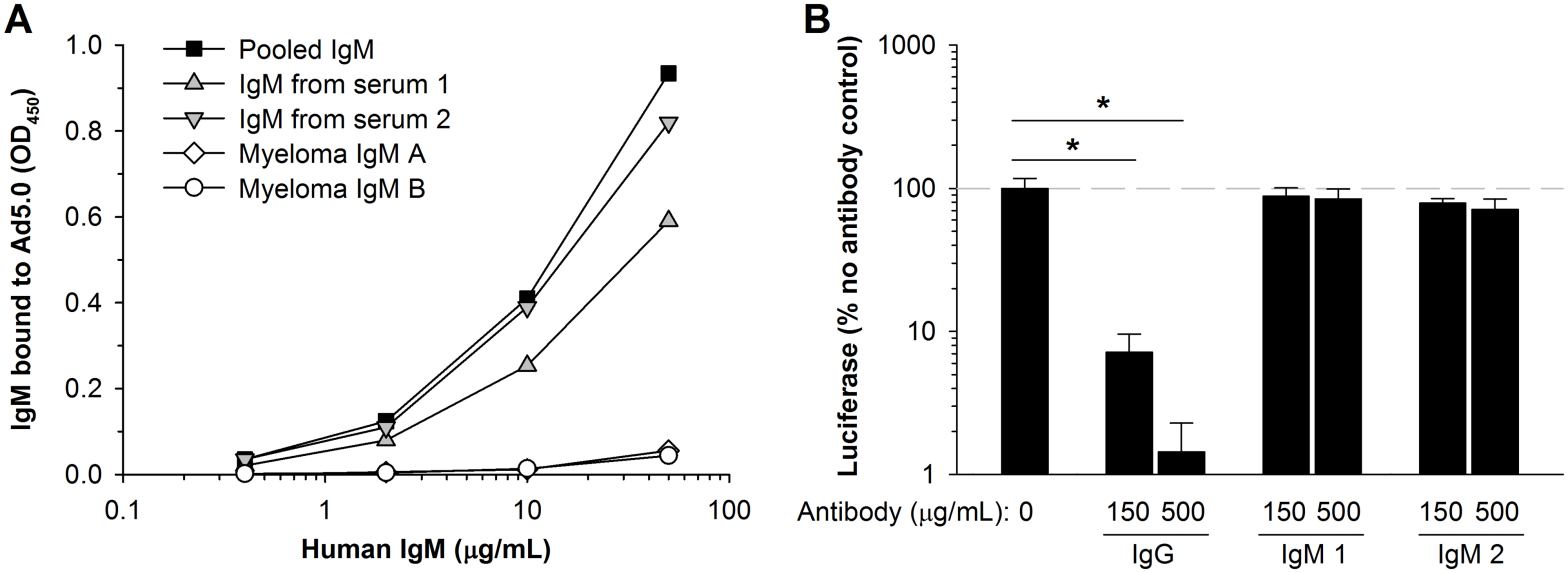
Human IgM binds to Ad5, but is insufficient for neutralization. (A) Binding of human IgM to Ad5.0-coated plates was assessed by ELISA. Sources of IgM were: IgM derived from pooled human serum, IgM from individual human sera 1 and 2, or IgM from sera of two IgM myeloma patients. (B) Infectivity of Ad5.0 vector after incubation with human IgG (from serum 18) or IgM (from sera 1 and 2) at either 150 or 500 μg/mL. Statistical significance was evaluated by one-way ANOVA, followed by the Holm-Sidak post-hoc test. * Groups are significantly different (*p* ≤ 0.05). Each bar represents the mean of 3 replicates ± S.D.

In mouse serum, neutralization is mediated by natural IgM that binds to the vector capsid and activates complement [10]. To determine the role that immunoglobulins play in neutralization by human serum, we purified IgM from two human samples (sera 1 and 2) that had low antibody-mediated neutralization activity (Fig 1A). Additionally, we purified IgG from a sample (serum 18) that had high antibody-mediated neutralization activity (Fig 1B). We have previously demonstrated that IgM purified from the serum of naïve mice is capable of binding to Ad5 [15, 18]. To determine if human IgM can bind Ad5, we performed an ELISA. We found that human IgM from individual sera exhibited substantial binding to Ad5.0, as did pooled human IgM that was obtained from a commercial supplier (Fig 2A). As an additional control, we included human IgM purified from the serum of patients with IgM myelomas, where essentially all of the IgM is from a monoclonal plasma cell tumor. Myeloma IgM from two commercial sources showed a poor ability to bind Ad5, suggesting that the ability of normal human IgM to interact with Ad is specific to a subset of IgM clones.

Mouse IgM binds to Ad5 but is insufficient on its own to neutralize vector [10]. Similarly, we found that human IgM had no neutralizing activity for Ad5 (Fig 2B). As a positive control, we found that IgG purified from serum 18 had significant neutralizing activity. Taken together, these data indicate that purified human IgM can bind Ad5 but not neutralize it, similar to previous results with purified IgM from naïve mice.

### Human antibodies can synergize with complement to neutralize Ad5, and FX protects against this neutralization

**Fig 3:**
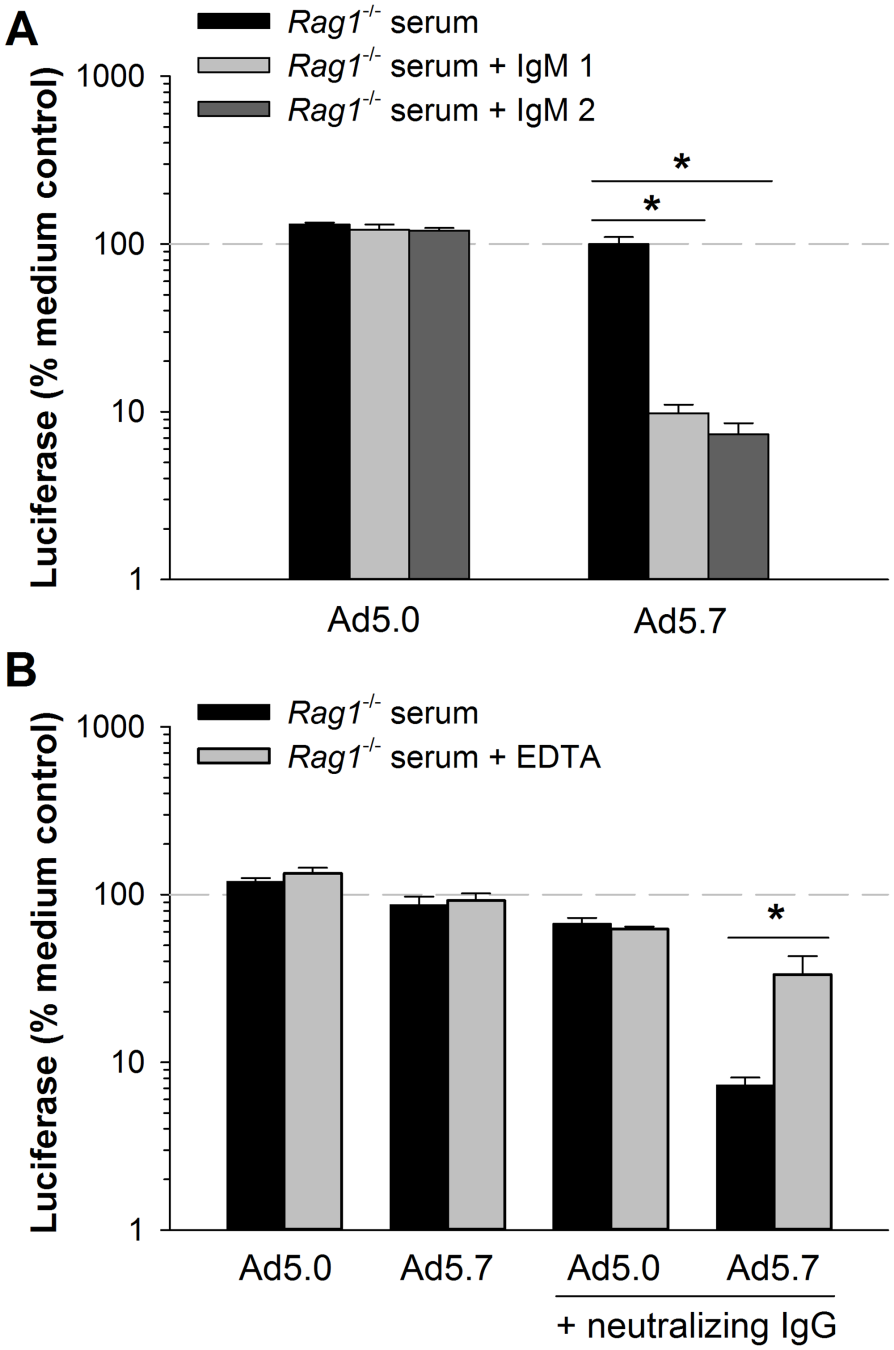
Human IgM and IgG enhance neutralization of vectors via complement, and FX protects vectors against this neutralization. Vectors were incubated with serum from *Rag1*^-/-^ mice, with or without the addition of human antibodies, and infectivity was assessed. (A) Infectivity of Ad5.0 (control vector) and Ad5.7 (non-FX-binding vector) after incubation with 150 μg/mL of human IgM (purified from sera 1 and 2) in the presence of mouse complement (serum from *Rag1*^-/-^ mice). (B) Infectivity of vectors after incubation with 50 μg/mL of human IgG (purified from serum 18) in the presence of active mouse complement, or after inactivating complement with 10 mM EDTA. The 100% reference lines indicate vector infectivity in medium without serum. Statistical significance was evaluated by one-way ANOVA, followed by the Holm-Sidak post-hoc test. * Groups are significantly different (*p* ≤ 0.05). Each bar represents the mean of 3 replicates ± S.D.

In mouse serum, mouse IgM can activate the classical complement pathway and neutralize vectors that are unprotected by FX [10], but it is not known whether IgM plays a similar role in human serum. In addition, it is completely unknown whether FX can protect against neutralization when complement is activated by IgG antibodies against Ad5. These questions are challenging to address experimentally in human serum, which contains both IgM and IgG. Therefore, we added purified human IgM and IgG separately to mouse complement (*Rag1*^-/-^ mouse serum, which lacks antibodies) and assessed the impact on vector infectivity. We found significant neutralization of the non-FX-binding vector Ad5.7 when it was incubated with human IgM plus *Rag1*^-/-^ serum (Fig 3A). In contrast, the Ad5.0 vector was not neutralized. This result indicates that human IgM can synergize with complement to neutralize Ad5 when the vector is not protected by FX, similar to previous findings with mouse IgM and complement [10].

Can coagulation factors also protect Ad5 against complement-mediated neutralization when complement activation is initiated by anti-Ad5 IgG? We addressed this question in experiments with human IgG plus mouse complement. We used human IgG purified from serum 18, which had high anti-Ad5 neutralizing antibodies, and mixed a low amount of this purified human IgG with mouse complement (*Rag1*^-/-^ serum). To distinguish direct neutralization of vector by anti-Ad5 IgG from secondary neutralization mediated by IgG-activated complement, each group was compared with a parallel group in which complement had been inactivated by EDTA. For the FX-binding vector Ad5.0, a low concentration of the human anti-Ad5 IgG had only modest neutralizing activity, and this neutralizing activity was unchanged by addition of EDTA (Fig 3B). Therefore, neutralization of Ad5.0 could be completely attributed to the IgG itself—there was no additional neutralization when complement was active. In contrast, we found that Ad5.7 was neutralized by IgG to a much greater extent when complement was active than when complement was inactivated by EDTA (Fig 3B). Thus, complement can enhance the ability of anti-Ad5 IgG to neutralize Ad5 vectors, but only when the vector is unable to bind FX. Taken together, these data indicate that FX significantly protects Ad5 from complement, regardless of whether the complement is activated by IgM or by IgG.

### Ad48 hexon confers resistance to mouse serum, but not to human serum

**Fig 4:**
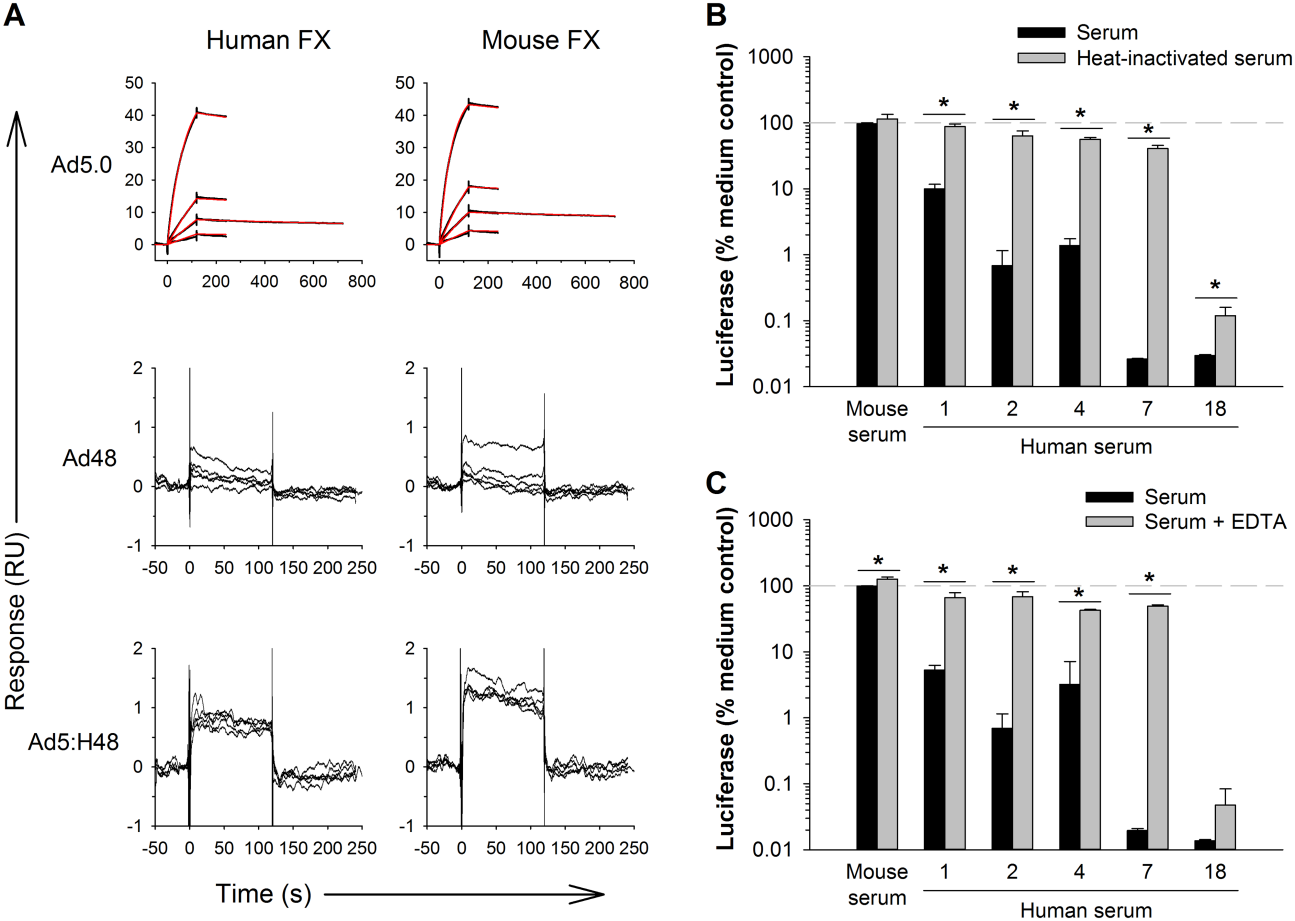
A hexon-chimeric vector with Ad48 hexon is neutralized by human serum, but not by mouse serum. The chime ric Ad5:H48 vector is a luciferase-expressing Ad5 vector in which the seven hypervariable regions of Ad5 hexon have been replaced by the corresponding regions of Ad48 hexon. (A) Representative surface plasmon resonance sensorgrams showing binding of FX to immobilized Ad5.0, Ad48 and Ad5:H48 (black lines). For Ad5.0, binding curves were fitted using a 1:1 binding model (red lines). The kinetic binding affinity (K_D_) of human FX for Ad5.0 was 0.82 nM, and the affinity of mouse FX for Ad5.0 was 0.42 nM. FX did not detectably bind to immobilized Ad5:H48 or Ad48. (B) Infectivity of Ad5:H48 vector following incubation with C57BL/6 mouse serum or individual human sera. To assess the role of complement, neutralization was also assessed with sera that had been heated to inactivate complement. (C) Infectivity of Ad5:H48 vector following incubation with serum in the presence or absence of EDTA to inactivate complement. The 100% reference lines indicate vector infectivity in medium without serum. Statistical significance was evaluated by t-test to compare the paired control and experimental groups, followed by the sequential Holm-Sidak procedure for multiple comparisons to control the experimentwise type I error rate at ≤ 0.05. * Groups are significantly different (*p* ≤ 0.05). Each bar represents the mean of 3 replicates ± S.D.

Although FX can bind to some Ad serotypes, many other serotypes such as Ad48 do not bind FX [5, 6]. Recently, Ma *et al*. [22] showed that mouse serum does not neutralize Ad48, regardless of the presence or absence of FX. This stability of Ad48 in mouse serum contrasts with Ad5, which is strongly neutralized by mouse serum in the absence of FX. The hypervariable regions of Ad48 hexon can be transferred to Ad5 vectors [25], and an Ad5 vector that contains the seven hypervariable regions from Ad48 shows complete resistance to neutralization by mouse serum, similar to the resistance of Ad48 itself [22]. In order to examine whether vectors with Ad48 hexon are also resistant to neutralization by human serum, we constructed a similar Ad5 vector containing the Ad48 hexon hypervariable regions (Ad5:H48) and examined its neutralization by serum. We used surface plasmon resonance assays to confirm that human FX and mouse FX were unable to bind to either Ad5:H48 or wild-type Ad48 (Fig 4A).

After a preliminary screen of heat-inactivated human sera to evaluate antibody-dependent neutralization of Ad5:H48, we chose to further investigate four human sera that showed the least antibody-dependent neutralization of Ad5:H48 (sera 1, 2, 4 and 7), along with one serum that showed high antibody-dependent neutralization (serum 18). Consistent with the report of Ma *et al*. [22], we found that Ad5:H48 was minimally affected by mouse serum (Fig 4B and 4C). Surprisingly, however, we found that Ad5:H48 was robustly neutralized by human sera. Neutralization of Ad5:H48 by human serum was greatly inhibited by heat inactivation of the serum (Fig 4B) or by EDTA (Fig 4C), indicating that complement plays a major role in the ability of human serum to neutralize Ad5:H48.

**Fig 5:**
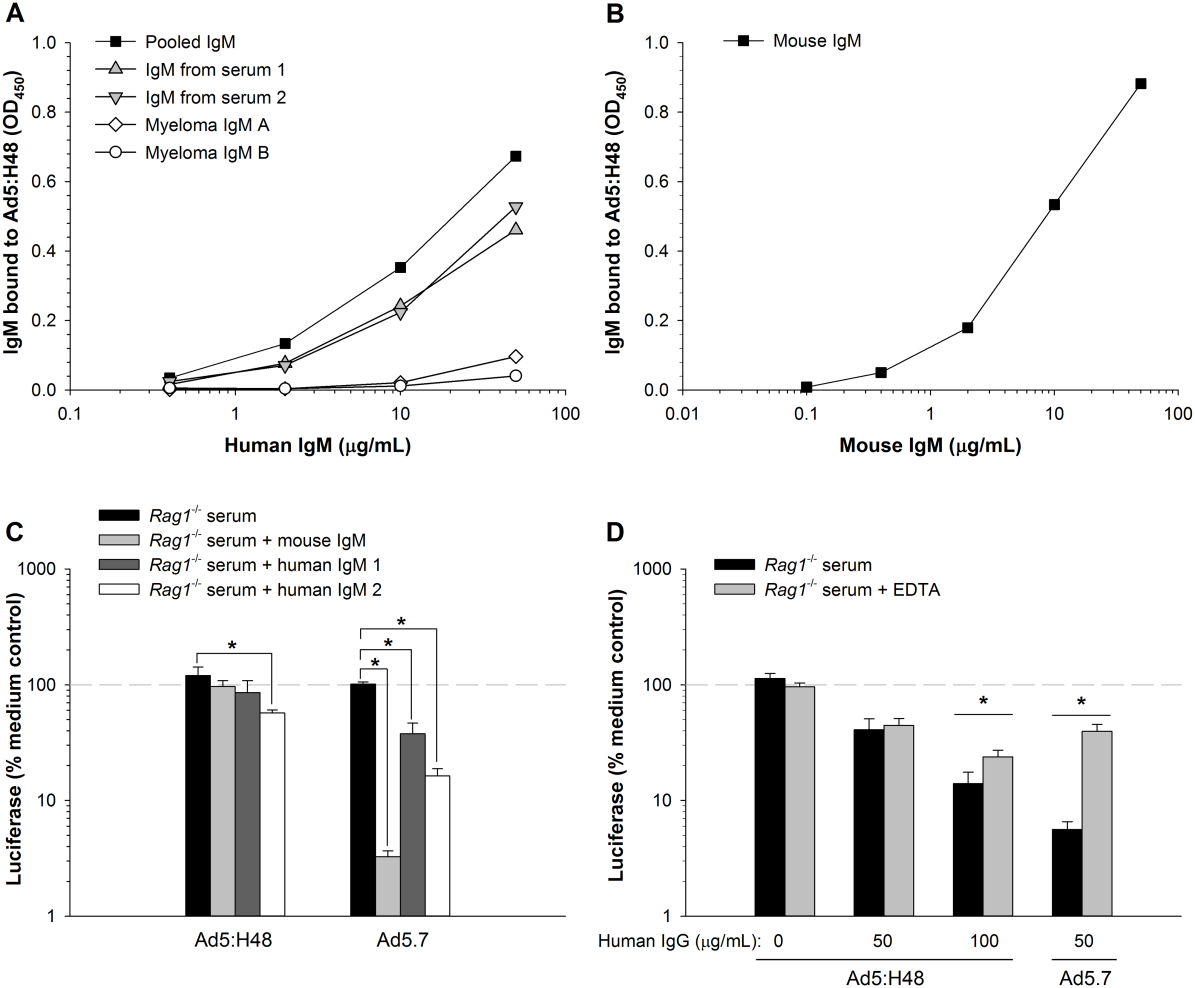
Interactions of human immunoglobulins and mouse complement with Ad5:H48. (A) Binding of human IgM to Ad5:H48 was assessed by ELISA, using the same sources of IgM as in Fig 2A. (B) Binding of mouse IgM to Ad5:H48. (C) Ad5:H48 and Ad5.7 were incubated with mouse complement (serum from *Rag1*^-/-^ mice), with or without addition of 300 μg/mL of mouse IgM or human IgM, and infectivity was assessed. (D) Infectivity of Ad5:H48 and Ad5.7 after incubation with human IgG (purified from serum 18) in the presence of active mouse complement, or after inactivating complement with 10 mM EDTA. The 100% reference lines indicate vector infectivity in medium without serum. Statistical significance was evaluated by one-way ANOVA, followed by the Holm-Sidak post-hoc test. * Groups are significantly different (*p* ≤ 0.05). Each bar represents the mean of 3 replicates ± S.D.

We attempted to determine whether human IgM and complement cooperate to neutralize Ad5:H48 in the same way that IgM and complement cooperate to neutralize Ad5.7. Human sera 1 and 2 had very low neutralizing activity for Ad5:H48 when the sera were heat-inactivated (Fig 4B), indicating that these sera had low amounts of neutralizing antibodies against Ad5:H48. IgM purified from these human sera showed good binding to Ad5:H48 (Fig 5A), as did mouse IgM (Fig 5B). When human IgM was mixed with *Rag1*^-/-^ serum as a source of complement, purified IgM from serum 2 (but not serum 1) showed statistically significant (but very modest) neutralization of Ad5:H48 (Fig 5C). Although this pattern of neutralization of Ad5:H48 was reproducible in an independent experiment, neutralization of Ad5:H48 was much weaker than neutralization of Ad5.7 under the same conditions (Fig 5C). Similarly, neutralization of Ad5:H48 by purified IgG from serum 18 was modestly enhanced by mouse complement, but the complement-dependent neutralization of Ad5:H48 was very weak compared to Ad5.7 (Fig 5D). Together, these experiments indicate that mouse complement is relatively poor at neutralizing Ad5:H48, even in the presence of human IgM or IgG. This finding demonstrates that human and mouse complement differ markedly in their ability to neutralize Ad5:H48.

## Discussion

Studies in animal models are essential for developing novel gene therapies and for predicting how gene therapy vectors will behave in humans. It is therefore important to understand the mechanisms that govern the biodistribution of gene therapy vectors and to determine whether these mechanisms differ between animal models and humans. Our data show that FX protects Ad5 in both mouse and human sera. In the absence of protection by FX, Ad5 vectors are robustly neutralized by both mouse and human serum, and this neutralization occurs via the same complement-dependent mechanism in both mouse and human sera. In contrast, we found that the hexon-chimeric vector Ad5:H48 was neutralized by human serum, but not by mouse serum. Thus, while mouse serum effectively mirrors both the biological mechanisms and outcomes that are seen for Ad5 in human serum, not all Ad vectors show a similar concordance between mouse and human serum.

### Similar interactions of Ad with mouse and human IgM

Previous reports indicate that serum proteins from mice and humans have comparable interactions with Ad5. Both mouse and human FX bind to Ad5 hexon with high affinity [6]. Likewise, both mouse and human IgM can bind to Ad5 [15, 17, 18, 26]. In the current study, we confirmed that human IgM can bind to Ad5, regardless of whether the IgM is purified from pooled human sera or from individual sera. We also performed experiments to further characterize human IgM and to extend our investigations to Ad5:H48.

We found that purified human IgM binds to Ad5 without substantial neutralizing activity, similar to previous findings with mouse IgM [10]. We also found that monoclonal IgM from two human myeloma sources had poor ability to bind Ad5 and Ad5:H48. These results parallel our prior observation that an anti-hepatitis B virus mouse monoclonal IgM antibody binds poorly to Ad5 [18]. Thus, our data show that both human and mouse IgM bind to Ad vectors with some degree of specificity.

We previously showed that polyreactivity (the ability of an antibody to bind to multiple antigens) is the key property that defines the ability of mouse IgM to bind to Ad5. Mouse IgM antibodies that are polyreactive can bind well to Ad5, while non-polyreactive mouse IgM binds poorly to Ad5 [18]. Although it is impossible to be certain that the human IgM samples that we purified from low-neutralizing sera are truly naïve natural antibodies, these human IgM samples behaved similarly to polyreactive natural IgM antibodies from naïve mice. It is well established that humans have polyreactive natural IgM [27]. Human natural IgM can bind to viruses that have never been encountered, as evidenced by the ability of human IgM to target viruses such as vesicular stomatitis virus that do not circulate in humans [28]. Given the broad reactivity of natural IgM against many different types of viruses, we were not surprised to find that both human and mouse IgM recognized not only Ad5, but also Ad5:H48. These findings imply that even humans who have never previously encountered Ad viruses would still have natural IgM antibodies that can bind to Ad vectors, just as naïve mice do.

### Similar effects of mouse and human complement on Ad5

We found that complement was essential for neutralization of Ad5 by naïve mouse serum and by human sera that contained low levels of neutralizing antibodies. In mouse serum, we have previously shown that Ad5 activates the antibody-mediated classical complement pathway when FX is unable to bind to the vector [10]. This complement activation in mouse serum (and subsequent neutralization of vector by mouse serum) requires natural IgM and complement proteins C1, C4 and C3. Although similar extensive studies of complement would be difficult to conduct in human sera, our data in human sera show clear parallels to mouse sera, with roles demonstrated for both human IgM and human complement. Similar to findings in naïve mouse serum, human sera with low neutralizing antibodies neutralized Ad5 vectors robustly when FX was blocked by X-bp or when hexon was mutated to prevent FX binding to the virus.

Although it has previously been reported by Mallery *et al*. [29] that human IgM can directly neutralize Ad5, we did not detect direct neutralizing activity either with human IgM in the current study or with mouse IgM in a previous study [10]. It is possible that the IgM-mediated neutralization of Ad5 that was detected by Mallery *et al*. [29] depends on IgM purity, cell lines or other assay conditions. We purified IgM from individual sera that had low pre-existing titers of antibodies against Ad5, and our purification strategy included both positive selection for IgM and negative selection to remove IgG. With these pure samples of human IgM, we found neutralization of Ad5 only when we added active complement, and even then we only saw neutralization if we prevented FX from binding to the vector.

One notable difference between mice and humans is that many humans have been exposed to adenovirus and have pre-existing IgG antibodies that can directly neutralize Ad vectors. Interestingly, we found that FX inhibits complement-mediated neutralization of Ad5 not only when complement is activated by human IgM, but also when complement is activated by human anti-adenoviral IgG (Fig 3B). Thus, although FX does not prevent direct IgG-mediated neutralization of Ad5 vectors, FX still plays a protective role by inhibiting the ability of IgG to trigger additional complement-mediated neutralization. This finding suggests that FX could offer Ad5 vectors at least some degree of protection against complement even in humans who have pre-existing anti-Ad5 immunity.

Similar to our findings, Duffy *et al*. [26] recently reported that human FX protects Ad5 vectors against neutralization by human sera, just as in mouse sera. However, only about half of the human sera in their study were capable of neutralizing Ad5 when FX was blocked. In contrast, we found that all twelve of our human sera were capable of neutralizing Ad5 when FX was blocked. Complement proteins are labile and complement activity can be degraded during blood collection, processing and storage [30]. Because active complement is essential for this type of neutralization [10], we speculate that variable complement potency among different serum samples may explain why Duffy *et al*. [26] found that a subset of their human sera lacked neutralizing activity.

### Ad5:H48 is neutralized by human serum, but not by mouse serum

Although Ad5 remains by far the most popular serotype for gene therapy studies with Ad vectors, investigators are increasingly turning to other human and animal Ad serotypes for both gene therapy and vaccine studies. The vector capsid determines interactions with both cellular receptors and blood proteins, and capsid proteins from alternative Ad serotypes may confer advantages for certain applications. For example, a chimeric Ad5 vector with Ad6 hexon has reduced clearance by macrophages due to decreased interaction with scavenger receptors on macrophages [16, 31].

In humans, IgG-mediated neutralization of Ad vectors is mediated mostly by antibodies against hexon, and therefore chimeric Ad5 vectors that contain hexons from rare Ad serotypes face lower levels of pre-existing neutralizing antibodies in humans [32, 33]. Ad48 is a rare human serotype, and surveys have found that hexon-chimeric Ad5 vectors containing the Ad48 hexon hypervariable regions are neutralized less frequently by heat-inactivated human sera [25, 34]. Chimeric Ad5 vectors with Ad48 hexon have been evaluated as gene therapy vectors, oncolytic viruses and vaccine vectors [35–39], including in a vaccine clinical study [40].

Ma *et al*. [22] demonstrated that mouse serum fails to neutralize both an Ad48 vector and a chimeric Ad5 vector with the Ad48 hexon hypervariable regions, and we observed the same for mouse serum. Surprisingly, however, we found that human serum robustly neutralizes Ad5:H48. Further investigation showed that both mouse and human IgM bind well to Ad5:H48, but that neutralization mediated by mouse complement is very weak, even in the presence of human antibodies.

More extensive experiments will be necessary to understand the mechanism for this striking species difference. It is conceivable that mouse complement proteins might have poor efficiency when interacting with Ad5:H48, even though mouse complement efficiently neutralizes Ad5.7. Another possibility would be if mouse serum (but not human serum) contains an inhibitor that protects Ad5:H48 from neutralization by complement. This hypothetical inhibitor cannot be FX, because FX is unable to bind to Ad5:H48, and indeed it has been shown that blocking FX with X-bp has no impact on the ability of vectors with Ad48 hexon to resist neutralization by mouse serum [22].

Regardless of the mechanism, the different susceptibility of Ad5:H48 to neutralization by mouse and human complement suggests a need for care when extrapolating studies of Ad48-based vectors from mice to humans. A human clinical trial of an intramuscular Ad5:H48 vaccine demonstrated development of immunity against the vector’s genetically-encoded antigen [40], so complement is certainly not an insurmountable barrier to the use of such vectors as vaccines in humans. However, more caution may be warranted when Ad48-based vectors are administered i.v., where the vector will rapidly encounter the complement system.

Relatively little work on human complement has been performed with non-Ad5 serotypes, and some of this past work was performed using citrate plasma [41]. While complement is active in citrate plasma, citrate reduces the Ca^2+^ concentration to a level that no longer supports binding of FX to Ad vectors [10], leading to an incomplete model system. Serum is a more appropriate model system for studies of Ad vectors because it allows activity of both complement and coagulation factors, but care must be taken to handle serum in a manner that preserves complement activity. A potentially even more realistic (but more complex) model system is human whole blood that has been anticoagulated with heparin. A vector with an Ad11-based capsid was tested in a human whole blood model and found to be much less inhibited by blood than an Ad5 vector [42]. This system has the advantage of evaluating not only interactions with antibodies, coagulation factors and complement, but also with blood cells. Blood cells in mice and humans show species-specific expression of viral receptors and complement receptors, and these differences can affect vector biodistribution *in vivo* [43, 44].

In sum, evaluating differences between mouse models and humans requires a detailed understanding of the underlying biology and careful attention to experimental conditions. We found that antibodies and complement from mice and humans interact similarly with Ad5 vectors, and we found that both mouse FX and human FX consistently inhibit neutralization of Ad5 vectors. These results suggest that mouse serum adequately models the most relevant features of the human complement system when performing studies with Ad5 gene therapy vectors. Surprisingly, we found that the hexon-chimeric vector Ad5:H48 was stable in mouse serum but neutralized by human serum, suggesting caution about relying solely on data from mouse models.

## Methods and materials

### Adenovirus vectors

All Ad vectors in this study have deleted E1 and E3 regions, and all vectors express luciferase under control of a CMV promoter. Ad vectors were constructed from plasmids as previously described [10], except that a modified version of the backbone plasmid pAdHM4-CMVL1 [45] was used. The vector Ad5.0 has a wild-type Ad5 capsid and is functionally equivalent to our previously-described vector AdCMVL1 [10]. The vector Ad5.7 contains four targeted mutations in the Ad5 hexon (I421G, T423N, E424S and L426Y) to prevent coagulation factor binding, and Ad5.7 is functionally equivalent to our previously-described vector AdHVR7 [10]. The vector Ad5:H48 was produced by a plasmid construction strategy that involved replacing each of the seven hypervariable regions of Ad5 hexon with the corresponding regions from Ad48 hexon. The resulting Ad5:H48 vector contains a chimeric Ad5/Ad48 hexon with a sequence exactly as described by Roberts *et al*. [25], plus the T342M hexon mutation that was described by Bruder *et al*. [46] to increase vector yield. Vectors were grown on 293 cells that were obtained directly from Qbiogene, Inc. (QBI-293A). Vectors were purified by CsCl ultracentrifugation, formulated and characterized as previously described [10]. Ad48 was obtained from the American Type Culture Collection (VR-1406) and was grown and purified in the same manner as the vectors.

### Human serum and antibodies

Thirty adult human sera were obtained from a supplier that collects and freezes sera in a manner that preserves complement activity (Innovative Research Inc., Novi, MI). After receipt, each 10 mL serum sample was thawed on ice and aliquoted at -80°C to avoid additional freeze-thaw cycles (which can be detrimental to complement activity). Antibodies were purified from selected individual sera as described below. ELISA control antibodies included IgM purified from pooled human serum (Athens Research Technology, #16-16-090713) and IgM purified from the serum or plasma of patients with IgM myelomas (Athens Research Technology #16-16-090713-M and SouthernBiotech #0158L-01). All human-derived materials in this study were commercially-available materials, with no individually-identifiable private information. Research using such materials does not meet the definition of human subjects research under US Code of Federal Regulations 45 Part 46.

### Mouse serum

Procedures using mice were carried out in strict accordance with FDA institutional guidelines and the standards contained in the Guide for the Care and Use of Laboratory Animals (The Guide, 8th Edition) of the National Institutes of Health. Animal protocols were approved by the FDA White Oak Consolidated Animal Care and Use Committee (protocols 2003–11 and 2004–16). Animal facilities were accredited by the Association for Assessment and Accreditation of Laboratory Animal Care International. All serum collection was performed as a terminal procedure on fully-anesthetized mice, and all efforts were made to minimize suffering.

Mouse serum for IgM purification was collected from BALB/c mice (Jackson Laboratories) due to the relatively high levels of IgM in this strain [18]. Sera for complement studies were collected from adult male C57BL/6J mice (Jackson Laboratories) and *Rag1*^-/-^ mice on the C57BL/6J background. *Rag1*^-/-^ mice lack B cells and therefore they do not have any antibodies [47]. Although human complement is stable when properly frozen, mouse complement is damaged by freezing. Therefore, for complement studies mouse serum was collected and kept on ice for use on the same day. Approximately 10 min before serum collection, mice were anesthetized by intraperitoneal injection of 150 mg of ketamine per kg of body weight and 30 mg of xylazine per kg of body weight. Blood was collected via cardiac puncture and immediately transferred to a serum separator tube containing a clot activator (BD). Clot formation was allowed to proceed for 30 min at room temperature, and then samples were spun in a table top centrifuge at full speed for 10 min at 4°C. Serum was carefully removed from the top layer of the separator tube and stored on ice until use.

### Neutralization assays

The day before each experiment, 293 cells were plated in collagen-treated 96-well plates at 1.5 x 10^4^ cells per well. Prior to seeding cells, rat tail collagen (ThermoFisher) was applied for 1 to 2 h at 2.5 μg/cm^2^ in 20 mM acetic acid, followed by a brief rinse with PBS.

On the day of each experiment, mouse serum was freshly prepared and aliquots of human serum were thawed on ice. To reduce variation in lipid levels among human sera, human sera were centrifuged at 10,000 x *g* for 2 min at 4°C, and the top layer was discarded. When heat-inactivated serum was required, serum was pretreated at 56°C for 30 min. Some neutralization reactions were performed with a final concentration of 10 mM EDTA per reaction to inactivate complement or 80 μg/mL X-bp [10, 24] to block FX. Neutralization reactions were prepared on ice, with Ad5 vectors added last. The final amount of vector in each 50 μL reaction was 1 x 10^8^ vector particles (vp) for a concentration of 2 x 10^9^ vp/mL, and the final concentration of serum was 80%. Negative control samples (baseline transduction) consisted of vector at 2 x 10^9^ vp/mL in serum-free DMEM supplemented with 2% globulin-free BSA (Sigma-Aldrich). For neutralization experiments using purified antibodies in the absence of serum, vectors were incubated with antibodies in DMEM with 2% globulin-free BSA.

Neutralization reactions were incubated at 37°C and 5% CO_2_ for 30 min and then placed on ice. Neutralization mixtures were diluted 2,000-fold in serum-free DMEM. 293 cells were rinsed once with PBS containing Ca^2+^ and Mg^2+^, and then 100 μL of diluted neutralization mixtures (containing 1 x 10^5^ vp) was added to each well for 2 h at 37°C. Following this incubation, the inoculum was replaced with medium containing 2% heat-inactivated FBS. After approximately 18 h, cells were rinsed with PBS and then lysed by incubation in 100 μL of lysis buffer (25 mM Tris-phosphate, pH 7.6; 2 mM EDTA;10% glycerol; 1% Triton X-100) at 4°C with 400 rpm orbital shaking for 30 min. Following lysis, plates were spun for 5 min at 1500 rpm at 4°C to pellet cellular debris. 20μL of lysate for each reaction was then transferred to a white-walled 96-well plate and injected with 100 μL of Luciferase Assay System Reagent (Promega) using the manufacturer’s protocol for a plate-reading luminometer (Glomax, Promega). Protein concentrations of lysates were determined by diluting 20 μL of lysate in 130 μL of water and then performing a Micro BCA Protein Assay (ThermoFisher) according the manufacturer’s instructions.

### Purification of antibodies from serum

IgM was purified from individual human sera or pooled BALB/c mouse serum using CaptureSelect IgM affinity matrix (POROS, ThermoFisher) on an AKTA Pure fast protein liquid chromatography system (GE), as previously described [48]. For human IgM, any contaminating IgG was removed using Protein G Sepharose beads (GE) according to the manufacturer’s instructions. Human IgM preparations were confirmed by ELISA to contain < 0.12% IgG contamination.

To purify human IgG, IgM-depleted serum fractions were loaded onto a HiTrap Protein G HP column (GE). Chromatography was conducted according to the manufacturer’s instructions. Following elution, IgG was buffer-exchanged to PBS and concentrated by ultrafiltration with an Amicon 10-kDa filter (MilliporeSigma).

### Antibody ELISAs

High absorption ELISA plates (Nunc MaxiSorp, ThermoFisher) were coated overnight at 4°C with antibody or vector diluted in bicarbonate/carbonate coating buffer (15 mM Na_2_CO_3_, 35 mM NaHCO_3_, pH 9.6). For Ad-binding ELISAs, plates were coated with 100 μL of Ad5.0 or Ad5:H48 at a concentration of 3 x 10^10^ vp/mL. For quantitation of IgM and IgG by ELISA, plates were coated with goat anti-human IgM (SouthernBiotech #2020–01) or goat anti-human IgG (SouthernBiotech #2040–01) at a concentration of 0.5 μg/mL.

Between all incubation steps, plates were washed in washing buffer (PBS with 0.9 mM Ca^2+^, 0.5 mM Mg^2+^ and 0.05% Tween-20). Blocking was performed with blocking buffer (PBS with Ca^2+^, Mg^2+^ and 1% globulin-free BSA) for 1 h at room temperature. All room temperature incubations were performed with 450 rpm orbital shaking. Antibodies were diluted in dilution buffer (PBS with Ca^2+^, Mg^2+^, 1% globulin-free BSA and 0.05% Tween-20) and incubated for either 2 h at room temperature or at 4°C overnight. For antibody quantitation ELISAs, human IgMλ isotype control (SouthernBiotech #0158–01) or human IgG isotype control (SouthernBiotech #0150–01) samples were included as standards.

Captured antibody was detected with either goat anti-human IgM antibody conjugated to HRP (SouthernBiotech #2020–05), goat anti-mouse IgM conjugated to HRP (SouthernBiotech #1021–05), or goat anti-human IgG antibody conjugated to HRP (SouthernBiotech #2040–05) diluted at 1:10,000 in dilution buffer and incubated for 1 h at room temperature. Colorimetric reactions were performed for 15 min using tetramethylbenzidine substrate (BD) and stopped using 2 N H_2_SO_4_. After brief mixing, optical density (OD) was measured at 450 nm using a plate reader (Glomax, Promega). Background signal (from non-specific antibody binding to plates) was removed for each sample by subtracting OD measured on uncoated wells from the OD measured on Ad-coated wells.

### FX binding assays

Surface plasmon resonance experiments were performed on a Biacore T200 system (GE Healthcare) using a protocol that was modified from Irons *et al*. [49]. Briefly, approximately 1,200 reference units of Ad5.0, Ad5:H48 or Ad48 were immobilized on a CM5 Biacore sensor chip. Running buffer was 10 mM HEPES, 150 mM NaCl, 1 mM CaCl_2_, 0.5 mM MgCl_2_, 0.1% (wt/vol) bovine serum albumin and 0.05% (vol/vol) polysorbate 20, pH 7.4. Human FX and mouse FX (Haematologic Technologies, Inc.) were diluted in running buffer at four concentrations (one of which was run in duplicate). The maximum concentrations of FX tested were 42 nM for Ad5.0 and Ad5:H48, and 212 nM for Ad48. The flow rate was 50 μL/min, and an extended dissociation time was used for one run to aid in obtaining an accurate off-rate. After each analyte, sensors were regenerated for 2 min with running buffer in which the CaCl_2_ and MgCl_2_ were replaced with 3 mM EDTA. Regeneration was followed by equilibration for 1 min in running buffer. Data were acquired at 1 Hz and globally fit to a 1:1 kinetic model using Biacore T200 Evaluation Software 3.0, with single referencing.

### Statistics

Statistical analyses were performed using SigmaPlot version 12.5 (Systat Software) and Excel (Microsoft). Data were log-transformed to equalize variances and evaluated using either one-way ANOVA or unpaired t-test, as indicated in the figure legends. For ANOVA, post-hoc pairwise comparisons were performed with the Holm-Sidak test, using SigmaPlot. For experiments in which we used multiple t-tests to evaluate pre-specified pairwise contrasts, *p* values from the t-tests were adjusted and evaluated using the sequential Holm-Sidak procedure [50]. This step-down procedure controls the experimentwise alpha at ≤0.05 and thereby prevents inflation of the type I error rate when using multiple t-tests in a single experiment.
